# The impact of additional resistance and balance training in exercise-based cardiac rehabilitation in older patients after valve surgery or intervention: randomized control trial

**DOI:** 10.1186/s12877-020-01964-3

**Published:** 2021-01-07

**Authors:** Egle Tamulevičiūtė-Prascienė, Aurelija Beigienė, Mark James Thompson, Kristina Balnė, Raimondas Kubilius, Birna Bjarnason-Wehrens

**Affiliations:** 1 Rehabilitation Department, Lithuanian University of Health Sciences, Eiveniu g. 2, LT-50161 Kaunas, Lithuania; 2 University of Zurich, Rämistrasse 71, CH-8006 Zürich, Switzerland; 3 Faculty of Medicine, Lithuanian University of Health Sciences, A. Mickevičiaus g. 9, LT-44307 Kaunas, Lithuania; 4 Institute of Cardiology and Sports Medicine, Department of Preventive and Rehabilitative Sport and Exercise Medicine, German Sport University Cologne, Am Sportpark Muengersdorf 6, 50933 Cologne, Germany

**Keywords:** Cardiac rehabilitation, Exercise training, Physical frailty, Valve surgery, TAVI

## Abstract

**Background:**

To evaluate the short- and mid-term effect of a specially tailored resistance and balance training provided in addition to usual cardiac rehabilitation (CR) care program in older patients after valve surgery/intervention.

**Methods:**

Single-center (inpatient CR clinic in Lithuania) randomized controlled trial. Two hundred fifty-two patients were assessed for eligibility on the first day of admittance to CR early after (14.5 ± 5.9 days) valve surgery/intervention between January 2018 and November 2019. Participants were coded centrally in accordance with randomization 1:1 using a computerized list. Control group (CG) patients were provided with usual care phase-II-CR inpatient multidisciplinary CR program, while intervention group (IG) patients received additional resistance and balance training (3 d/wk). Patients participated in a 3-month follow-up. Main outcome measures were functional capacity (6 min walk test (6MWT, meters), cardiopulmonary exercise testing), physical performance (Short Physical Performance Battery (SPPB, score) and 5-m walk test (5MWT, meters/second)), strength (one repetition maximum test for leg press), physical frailty (SPPB, 5MWT).

**Results:**

One hundred sixteen patients (76.1 ± 6.7 years, 50% male) who fulfilled the study inclusion criteria were randomized to IG (*n* = 60) or CG (*n* = 56) and participated in CR (18.6 ± 2.7 days). As a result, 6MWT (IG 247 ± 94.1 vs. 348 ± 100.1, CG 232 ± 102.8 vs. 333 ± 120.7), SPPB (IG 8.31 ± 2.21 vs. 9.51 ± 2.24, CG 7.95 ± 2.01 vs. 9.08 ± 2.35), 5MWT (IG 0.847 ± 0.31 vs. 0.965 ± 0.3, CG 0.765 ± 0.24 vs 0.879 ± 0.29) all other outcome variables and physical frailty level improved significantly (*p* < 0.05) in both groups with no significant difference between groups. Improvements were sustained over the 3-month follow-up for 6MWT (IG 348 ± 113 vs. CG 332 ± 147.4), SPPB (IG 10.37 ± 1.59 vs CG 9.44 ± 2.34), 5MWT (IG 1.086 ± 0. 307 vs CG 1.123 ± 0.539) and other variables. Improvement in physical frailty level was significantly more pronounced in IG (*p* < 0.05) after the 3-month follow-up.

**Conclusion:**

Exercise-based CR improves functional and exercise capacity, physical performance, and muscular strength, and reduces physical frailty levels in patients after valve surgery/intervention in the short and medium terms. SPPB score and 5MWT were useful for physical frailty assessment, screening and evaluation of outcomes in a CR setting. Additional benefit from the resistance and balance training could not be confirmed.

**Trial registration:**

NCT04234087, retrospectively registered 21 January 2020.

## Background

Exercise-based cardiac rehabilitation (CR) is generally recommended for patients after valve replacement or intervention [1–4]. Scientifically, however, this recommendation is based above all on the well-documented positive prognostic effect of exercise training in cardiac diseases in general, and in coronary heart disease patients in particular [1]. The few studies that have evaluated CR effectiveness after surgical valve replacement or intervention report results from heterogenic CR measures, with regard to patient profile [5], type of intervention [6], and well as CR duration [6, 7]. The results of a Cochrane review (2 RCT, *N* = 148, 3–6 months CR) [5] confirm the lack of evidence in this field. Sufficient evidence-based data on adverse events, mortality, quality of life, symptoms and reversible left ventricular remodeling are not yet available [5]. A recently published large American cohort study demonstrated CR participation to be associated with reduction in hospital admissions and mortality rate within the first year after CR completion [8]. However, there are no generally accepted and evaluated standards for the content, mode, volume, and intensity of exercise-based CR after valve surgery or intervention [1, 2]. Furthermore, there is an ongoing discussion on how to assess frailty, as well as other important CR outcomes that might differ from those in other CR cohorts [9–11]. Using comprehensive frailty evaluation including physical, cognitive, nutritional and disability measures, Eichler et al. [12] showed positive short-term CR effects on frailty for transcatheter aortic valve implantation (TAVI) patients. Adding resistance/balance training to usual CR care led to a significant increase in time to Timed up and go (TUG) test in older patients after bypass surgery [13]. While several small studies demonstrate improvements in Short Physical Performance Battery test (SPPB) score in frail patients as a result of long-term exercise training [14–16], no other studies have evaluated the short or medium term CR impact on physical frailty measured by SPPB score or gait speed exclusively in patients after valve surgery/intervention.

The aim of our study was to evaluate the short- and mid- term effect of additionally provided (3 sessions per week), specially tailored resistance/balance training during 3-week inpatient CR in older patients after valve surgery/intervention, specifically its impact on functional capacity, physical performance (primary outcome) exercise capacity, muscular strength and prevalence and/or symptoms of physical frailty.

## Methods

### Study design

This study was designed as a randomized controlled (allocation ratio 1:1) single-center interventional study. It was conducted according to the principles of good clinical practice.

### Study participants

Inclusion period was January 2018 to November 2019. During this period, 252 post-valve surgery or valve intervention patients were admitted to the Kulautuva rehabilitation center at Lithuania University of Health Sciences Hospital’s for phase-II inpatient CR. Inclusion criteria were: age ≥ 65 years; the ability to start CR within ≤4 weeks after surgery/intervention; 6-min walk distance (6-MWD) ≥100 - ≤350 m to ensure homogeneity of the study population; and patient consent to participation in the study. Exclusion criteria were: heart failure New York Heart Association (NYHA) Class IV; hemoglobin < 9 g/dL; wound healing disorders; cognitive and/or mental disorders; linguistic deficits; as well as exercise-limiting comorbidities (primarily orthopedic, neurological conditions) that would exclude the patients from participating in CR according to study protocol. Trial was ended after calculated sample size + 20% was reached.

### Study assessment

Assessment times were: before randomization (admittance to CR) (T0); at CR-completion (T1); and at three months after CR completion (T2). All assessments were blinded and performed by certified staff members that were not involved in clinical care (two medical doctors with specialization in cardiology, one physiotherapist and one nurse). The assessment included medical history (i.e. cardiac diagnosis, cardiovascular risk factors, concomitant diseases, as well as at T2 clinical course, events and hospitalization since last examination), a clinical examination including echocardiography and cardiopulmonary exercise testing (CPET), and measurements of anthropometric data were performed. The CPET was performed on a cycle ergometer using a ramp protocol starting with 25 watt (W) and increasing 12.5 W per minute until subjective exhaustion or occurrence of abort criteria. Peak workload (W and W/kg) and peak oxygen consumption (VO_2_ ml/kg /min) were measured. Furthermore, a six-minute walk test (6MWT) was performed according to the American Thoracic Society guidelines [17] to evaluate functional capacity. One repetition maximum (1RM) was assessed for leg exertion (leg press) using a resistance machine (HUR, Finland).

We used SPPB (0–7 points – frail, 8–9 pre-frail, 10–12 – robust) [10, 18–20] and gait speed test, using 5MWT (≤0,69 m/s – frail, 0,7–0,99 m/s – pre-frail, ≥1,0 m/s – robust) [10, 19–21] to evaluate physical frailty level [10, 14, 15, 18, 20].

### Study interventions

All study participants attended standardized 20-calendar-day inpatient multidisciplinary phase-II CR including patient education, diet counseling, psychological support, risk factor management as well as individually dosed and adapted exercise training. Duration and intensity of the training session were individually adapted based on clinical and functional status.

The usual care supervised exercise program (Appendix 1) included: 1) *continuous endurance training* on cycle ergometers (6 sessions a week). Every session included warm-up (< 50% target intensity 2 min, gradually increasing load 1–10 W/min up to target intensity within 5–10 min); exercise phase (100% of the target intensity (30–50% watt_max_ or 60–70% maximal heart rate (HR_max_)), starting with > 5 min and gradually lengthening up to 30 min); cool down with gradual reduction of the load within 3 min); 2) *aerobic dynamic gymnastics in sitting and/or standing* position (30 min, 5 days/week); 3) *respiratory muscle training* (7 days/week, for 15 min) using lung exerciser (Respiprogram, Germany).

Subjects randomized to the IG attended the 20 day inpatient usual care exercise training sessions with the CG patients and then had additional exercise sessions (Appendix 2) including resistance and balance training three sessions/week. These group training sessions were delivered by a physiotherapist in individually tailored small-group (3 patients) training sessions, unlike usual care that is provided in groups of up to 7 patients.

The resistance training was started no earlier than on the third CR day. The focus was on posterior pelvic muscles, posterior and superficial tibia muscles, posterior and anterior thigh muscles, including 4–6 exercises using free weights, resistance bands, gravity-resisted exercises and other machines (HUR, Finlad), as well as other exercises. The resistance training started with low intensity (< 30% 1-RM, rate of perceived exertion (RPE) ≤ 11, 5–10 repetitions) and was gradually increased up to moderate intensity (30–50% and up to 60% 1-RM, RPE 12–13, 8–15 repetitions) performing 3 sets with a 3 min rest between sets, if tolerated.

The balance training included exercises to improve static and dynamic balance ability. It was performed on 2–3 days/week for 10–15 min. The complexity of the balance exercises was selected and incremented individually by changing the standing position (standing on both legs/standing upright/standing on one leg), the base on which the stands were performed (flat/uneven) and/or using unstable surfaces. Furthermore, if tolerated, the visual information was varied (open/closed eyes) and/or additional tasks performed while balancing.

After completion of the CR, the IG participants were encouraged to continue exercise training at home according to physiotherapist recommendations. They received a telephone call every two weeks (6 calls during 12 weeks follow up period) and were also asked to answer questions regarding their health and physical activity. The control group did not receive any follow-up telephone calls.

### Primary outcomes

The short- (at completion of CR) and medium-term (3 months after CR completion) effectiveness of additional resistance and balance training compared to usual care CR to improve functional capacity (6MWT) and physical performance (SPPB and 5MWT).

### Secondary outcomes

The short- and medium-term effectiveness of additional resistance and balance training compared to usual care CR to improve exercise capacity (peak work load, peak VO2) and muscular strength (1RM).

The short- and medium-term effectiveness of the additional resistance and balance training compared to usual care CR on prevalence and/or symptoms of physical frailty.

### Ethics

The study protocol was approved by *Kaunas* Regional Biomedical Research *Ethics Committee (*Nr. BE-2-39, BE-2-57*).*

### Sample size

The sample size was calculated as a function of the expected change in the results of the SPPB score. In order to detect a 1 standard deviation difference in SPPB score between the arms, we calculated we would need 91.4 evaluable patients under the assumption of a two-sided type I error of 5% and a power of 80% (t-test). Rounding up and accounting for an expected loss to follow-up of 5% (in terms of missing primary outcome data) implied that we would require a sample size of ~ 96 patients. Our final sample size was about 20% larger than this computed requisite sample size.

### Randomization and data management

Subjects eligible for the trial were randomly assigned to two groups. Study investigators enrolled participants and coded them centrally in accordance with randomization 1:1 to IG or CG using a computerized list. All assessors were blinded to the randomization.

### Statistical analysis

All analyses were performed in the intention-to-treat population. Continuous and categorical variables are presented by mean, standard deviation, absolute, and relative frequencies. Chi-square tests and t-tests were used to test for baseline differences between groups.

Multivariate analysis of variance with repeated measurements was used for statistical analyses of time-, group-, and treatment-related changes and differences, with *p* < 0.05 considered as significant. We know that the missing data was generated by a missing at random process due to some sessions accidentally not being recorded, hence the missing values were not deemed biasing and not imputed. Results were analyzed in two stages: first, global means were interpreted and, second, the marginal scores were compared if the respective *p*-value was significant. No alpha adjustment was required for multiple hypothesis testing. Adjustment for the respective scores was included to account for heterogeneity amongst the various participants. Missing values were not imputed since the chosen estimation methods were considered to be robust for random missing entries. Physical frailty level differences between groups were tested using Chi-square and Fisher’s exact test (due to small cell counts). All tests were performed two-sided with *p*-values less than 0.05 indicating significance. Statistics were calculated using R version 3.5.1 and Jamovi 1.0.8.

## Results

### Study group

A sample of 252 patients was assessed for eligibility by a medical doctor (ETP, AB) on the first day of admittance to the rehabilitation hospital during their first clinical evaluation. One hundred sixteen patients (76.1 ± 6.7 years, 58 (50%) male) who fulfilled the inclusion criteria were randomized to the intervention (IG, *n* = 60) or a control group (CG, *n* = 56). There were 44 (37,9%) participants after isolated/combined valve surgery, 47 (40,5%) after valve and bypass surgery and 25 (21,6%) after TAVI. Aortic valve surgery was the most common (67, 57,8%) and its incidence differed among the study groups (IG 40 (66,7%) vs CG 27 (48,2%), *p* = 0.044).

All patients participated in a 20-calendar-day inpatient phase-II CR (18.6 ± 2.7 days on average) early after valve surgery/intervention. Admission to CR was 14.5 ± 5.9 days post-surgery/intervention. Groups were generally well matched except there were significantly more people with aortic valve surgery in the IG, and more people with chronic obstructive pulmonary disease in the control group, although no significant differences in VO_2_ or 6MWT were evident between groups. Patient baseline characteristics are summarized in Table 1.

**Table 1:** Patient baseline characteristics, differentiated between control and intervention groups

	All *N* = 116	IG n = 60	CG n = 56	*p*-value
Women	58 (50%)	30 (50%)	28 (50%)	1.0
Men	58 (50%)	30 (50%)	28 (50%)	
Age, years M ± SD	76.1 ± 6.6	75.9 ± 6.6	76.4 ± 6.6	0.711
Height, m, M ± SD	1.67 ± 0.1	1.67 ± 0.1	1.67 ± 0.1	0.915
Weight, kg, M ± SD	78 ± 14.7	78.2 ± 13.9	77.9 ± 15.5	0.921
Body mass index, kg/m^2^ M ± SD	27.7 ± 4.3	27.9 ± 3.9	27.6 ± 4.9	0.643
LV EF (%), M ± SD	45.9 ± 9.0	46.9 ± 8.6	44.9 ± 9.4	0.232
Post-surgery, days, mean M ± SD	14.5 ± 5.9	14.3 ± 5.7	14.8 ± 6.3	0.611
**Cardiovascular risk factors, n (%)**				
Hyperlipidemia	74 (63.8%)	37 (61.7%)	37 (66.1%)	0.622
Arterial hypertension, n (%)	96 (82.8%)	52 (86.7%)	44 (78.6%)	0.249
Diabetes mellitus, n (%)	23 (19.8%)	9 (15%)	14 (25%)	0.177
**Surgery/intervention**				
Isolated/combined valve surgery n (%)	44 (37.9%)	21 (35%)	23 (41.1%)	0.501
Valve and bypass surgery, n (%)	47 (40.5%)	28 (46.7%)	19 (33.9%)	0.163
Aortic valve surgery, n (%)	67 (57.8%)	40 (66.7%)	27 (48.2%)	**0.044**
Mitral valve surgery, n (%)	22 (19%)	9 (15%)	13 (23.2%)	0.259
Tricuspid valve surgery, n (%)	26 (22.4%)	12 (20%)	14 (25%)	0.519
TAVI n (%)	25 (21.6%)	11 (18.3%)	14 (25%)	0.383
**New York Heart Association Class (NYHA), n (%)**				
NYHA II	45 (38.8%)	25 (41.7%)	20 (35.7%)	0.511
NYHA III	71 (61.2%)	35 (58.3%)	36 (64.3%)	
**Atrial fibrillation, n (%)**				
Paroxysmal	3 (2.6%)	1 (1.7%)	2 (3.6%)	0.518
Persistent	28 (24.1)	13 (21.7%)	15 (26.8%)	0.520
Permanent	28 (24.1%)	13 (21.7%)	15 (26.8%)	0.520
**Physical capacity**				
Peak work load (Watt/kg) M ± SD	0.87 ± 0.26	0.92 ± 0.85	0.83 ± 0.19	0.283
peak VO_2_ (ml/kg/min) M ± SD	11.3 ± 3.56	11.2 ± 4.38	11.3 ± 2.81	0.909
Six-minute walking distance (m) M ± SD	239 ± 9.6	246 ± 93.5	231 ± 98.8	0.437
**Physical frailty level according to SPPB score n, (%)**				
Frail (0–7)	35 (36.8%)	20 (37.7%)	15 (35.7%)	0.839
Pre-frail (8, 9)	28 (29.5%)	13 (24.5%)	15 (35.7%)	0.235
Robust (10–12)	31 (33.7%)	20 (37.7%)	11 (26.2%)	0.233
**Physical frailty level according to five meters walking test (5MWT) n, (%)**				
Frail (≤0,69 m/s)	36 (39.1%)	20 (38.5%)	16 (40%)	0.881
Pre-frail (0,7–0,99 m/s)	38 (41.3%)	20 (38.5%)	18 (45%)	0.528
Robust (≥1,0 m/s)	18 (19.6%)	12 (23.5%)	6 (15%)	0.311
**Comorbidities, n (%)**				
Chronic obstructive pulmonary disease	9 (7.8%)	1 (1.7%)	8 (14.3%)	**0.011**
Degenerative joint disease	9 (7.8%)	4 (6.7%)	5 (8.9%)	0.649
Cancer	6 (5.2%)	2 (3.4%)	4 (7.2%)	0.328
**Medication, n (%)**				
Platelet inhibitor	51 (44%)	23 (38.3%)	28 (50%)	0.206
Warfarin	95 (81.9%)	49 (81.7)	46 (82.1%)	0.947
Beta-receptor-blocker	108 (93.1%)	55 (91.7%)	53 (94.6%)	0.527
Renin-angiotensin-aldosterone system inhibitors	102 (87.9%)	53 (88.3%)	49 (87.5%)	0.948
Diuretic	106 (91.4%)	53 (88.3%)	53 (94.6%)	0.226
Statin	58 (50%)	33 (55%)	25 (44.6%)	0.265
Oral antidiabetic medication	13 (11.2%)	4 (6.7%)	9 (16.1%)	0.109
Insulin	5 (4.3%)	1 (1.7%)	4 (7.1%)	0.147

Abbrevations: *IG* intervention group, *CG* control group, *n* number, *M* mean, *SD* standard deviation, *m* meters, *kg* kilograms, *ml* mililiters, *min* minutes, *SPPB* short physical performance battery test, *LV EF* left ventricular ejection fraction, *TAVI* transcatheter aortic valve implantation

### Cardiac rehabilitation effect

#### Short-term results

**Fig. 1:**
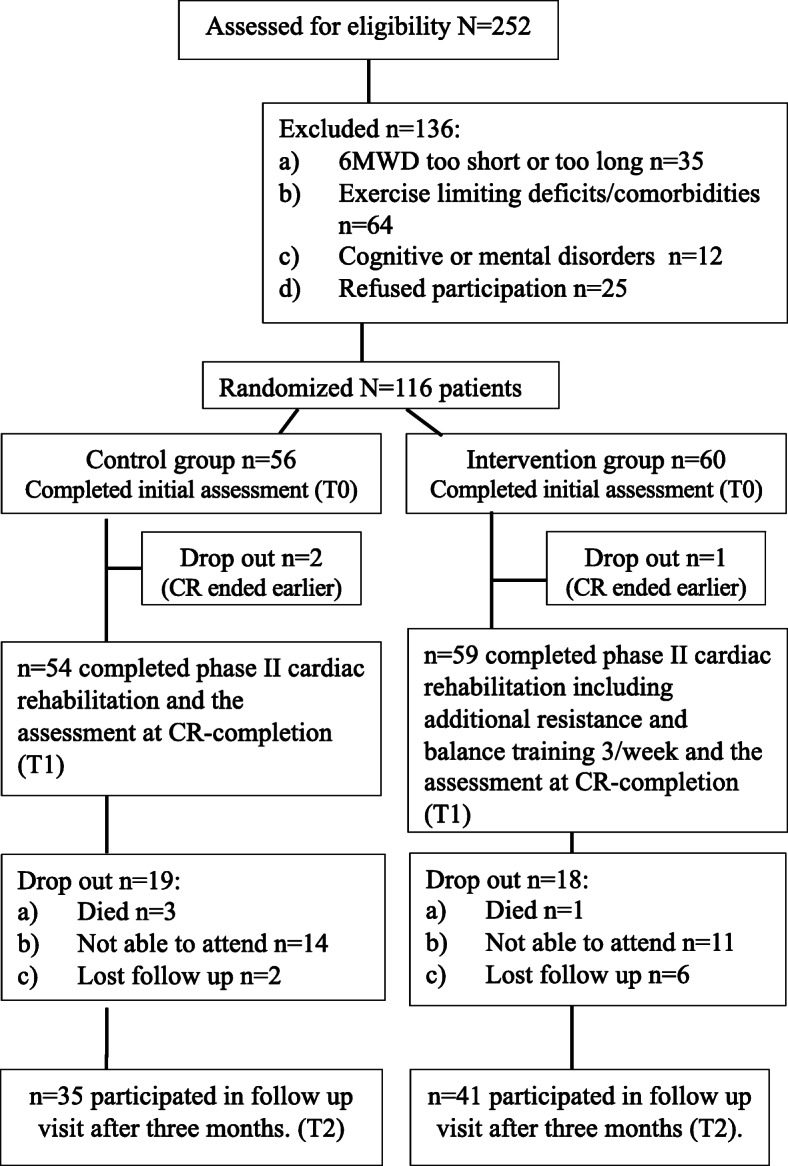
Study flow chart

Of the 116 randomized patients 113 (97.4%; CG *n* = 54, IG *n* = 59) completed the CR and the T1 assessment as planned. CR was ended prematurely in two CG subjects (transfer back to cardiac surgery department due to ventricular tachycardia episode and hematoma), and one patient in the IG ended the CR before the scheduled time due to personal reasons (fig. 1).

IG patients stayed at CR setting one day longer on average (*p* = 0.0021) and attended more exercise training sessions (*p* = 0.001) (Table 2). Additional exercise sessions were provided and supervised by an experienced physiotherapist in a small groups of up to 3 patients. IG patients participated in 6.1 ± 1.2 additional exercise sessions on average, and 28 (51,85%) of them performed the training program as intended (attended sessions and reached intensity as planned).

**Table 2:** Short-term CR effect on functional capacity, frailty-scores, exercise capacity and muscular strength. Multivariate analysis of variance with repeated measurements and Chi-square tests were used to detect changes between intervention and control groups. T0 = assessment at admittance to CR, T1 = assessment at CR-completion

	Intervention group (*n* = 59)			Control group (*n* = 54)			*p*-value		
Functional capacity	T0 M ± SD	T1 M ± SD	D T1-T0 M ± SD	T0 M ± SD	T1 M ± SD	D T1-T0 M ± SD	Time	Group	Interaction
6MWD (m)	247 ± 94.1	348 ± 100.1	108 ± 73.3	232 ± 102.8	333 ± 120.7	104 ± 90.4	< 0.01	0.960	0.428
SPPB (score)	8.31 ± 2.21	9.51 ± 2.24	1.18 ± 1.51	7.95 ± 2.01	9.08 ± 2.35	1.14 ± 1.78	< 0.01	0.847	0.380
5MWT (m/s)	0.847 ± 0.31	0.965 ± 0.32	0.117 ± 0.21	0.765 ± 0.24	0.879 ± 0.29	0.114 ± 0.19	< 0.01	0.946	0.204
Work load (watt)	70.5 ± 24.9	76.4 ± 26.7	7.25 ± 4.5	67.8 ± 16.6	81.8 ± 24.5	14 ± 14.6	< 0.01	0.121	0.868
Work load (watt/kg)	0.94 ± 0.30	1.02 ± 0.32	0.08 ± 0.06	0.85 ± 0.17	1.04 ± 0.27	0.18 ± 0.09	< 0.01	0.114	0.690
PeakVO_2,_ (ml/kg/min)	12.8 ± 5.6	13.3 ± 3.5	1.47 ± 5.4	11.6 ± 2.9	13.3 ± 3.5	0.37 ± 3.4	0.097	0.929	0.451
1RM for leg extension (kg)	46.6 ± 17.5	59.5 ± 21.5	10.1 ± 11.9	50.5 ± 20.9	53.5 ± 21.1	8.7 ± 9.7	< 0.01	0.606	0.160
CR duration, days, mean M ± SD	19.2 ± 1.9			18.2 ± 2.6			**0.021**		
Exercise training sessions, mean M ± SD	36.4 ± 2.84			28.7 ± 4.09			**0.001**		
Frailty level	Intervention group (*n* = 59)			Control group (*n* = 54)			*p*-value		
Frailty level according to SPPB score	T0 (n, (%))	T1 (n, (%))		T0 (n, (%))	T1 (n, (%))		Time		Group
Frail	20 (40.8%)	14 (28.6%)		14 (37.8%)	11 (29.7%)		< 0.01		0.13
Pre-frail	11 (22.4%)	7 (14.3%)		13 (35.1%)	8 (21.6%)				
Robust	18 (36.7%)	28 (57.1%)		10 (27%)	18 (48.6%)				
Frailty level according to 5MWT	T1 (n, (%))	T2 (n, (%))		T1 (n, (%))	T2 (n, (%))		Time		Group
Frail	19 (40.4%)	10 (21.7%)		15 (41.7%)	11 (30.6%)		< 0.01		0.12
Pre-frail	17 (36.2%)	19 (41.3%)		16 (44.4%)	16 (44.4%)				
Robust	11 (23.4%)	17 (37%)		5 (13.9%)	9 (25%)				

Abbrevations: *T0* assessment at admittance to *CR, T1* assessment at CR completion, *n* number, *M* mean, *SD* standard deviation, *6MWD* six minute walking distance, *m* meters, *SPPB* short physical performance battery test, *5MWT* five meters walking test, *m/s* meters per second, *kg* kilograms, *ml* mililiters, *min* minutes, *1RM* one repetition maximum

The main short-term CR results are summarized in Table 2. As a result of the CR, all measured parameters, with exception of peak VO_2_, improved significantly in both groups. The results revealed no statistically significant difference between groups and no significant intervention effect (*p*-value interaction).

Physical frailty level was assessed for 86 (76.1%) of 113 patients who finished phase-II CR. SPPB score and 5MWT indicated a comparable number of patients as being frail in the T0 and T1 evaluations performed: SPPB: T0 36.8%; T1 24%; 5MWT: T0 39.1%; T1 29%. On the other hand, a comparatively high number of patients was categorized as pre-frail using the 5MWT (41.3% vs 29.5%) and fewer as robust (19.6% vs. 33.7%) compared to SPPB score. The calculated physical frailty level improved significantly in both groups, but no significant group or interaction effects were demonstrated (Table 2).

#### Medium-term CR results

Of the 113 randomized patients that completed the CR and the T1 assessment as planned, 76 (67.3%; CG *n* = 35; IG *n* = 41) participated in the three-month follow-up visit. The reasons for not participating were: in four cases death; 25 cases refused to participate, or were not able to attend the follow-up visit because of distance from the center, lack of social support or health issues. In total, 8 patients were lost to follow-up (fig. 1). The main medium-term CR results are summarized in Table 3. The medium-term CR results revealed significant changes in all measured parameters in both groups, but no significant group or interaction effects were demonstrated.

**Table 3:** Medium-term CR effect on functional capacity, frailty-scores, exercise capacity and muscular strength. Multivariate analysis of variance with repeated measurements and Fisher’s exact tests were used to detect changes between intervention and control groups

	Intervention group *n* = 41						Control group *n* = 35						*p*-value		
Functional capacity	T0 M ± SD	T1 M ± SD	T2 M ± SD	D T1-T0 M ± SD	D T2-T1 M ± SD	D T2-T0 M ± SD	T0 M ± SD	T1 M ± SD	T2 M ± SD	D T1-T0 M ± SD	D T2-T1 M ± SD	D T2-T0 M ± SD	Time	Group	Inter-action
6MWT (m)	251± 9.1	366± 104.0	348± 113.1	115.5± 82.3	18.6± 74.9	96. ± 98.1	260± 106.4	357± 123.3	332± 147.4	102.9± 106,3	21.2± 106.6	81.6± 123.9	< 0.01	0.635	0.856
SPPB (score)	8.47± 2,37	9.8± 2.3	10.37± 1.59	1,33± 1.71	0.567± 1.63	0.9± 2.29	8.56± 2.03	9.00± 2.37	9.44± 2.34	0.438± 1.59	0.438± 2.22	0.875± 2.25	< 0.01	0.346	0.185
5MWT (m/s)	0.907± 0.341	1.049± 0.392	1.086 ± 0. 307	0.14± 0.22	0.03± 0.28	0.18± 0.28	0.761± 0.267	0.878± 0.321	1.123± 0.539	0.117± 0.136	0.244± 0.427	0.36± 0.5	< 0.01	0.08	0.360
Peak work load (watt)	70.3± 25.9	78,1± 27	93.6± 42.8	9± 27.1	19.5± 24.5	28.5± 32.7	71.5± 17.3	87.0± 25.5	91.8± 21.7	14.6± 15.7	5.5± 13.0	20.1± 13.9	< 0.01	0.289	0.385
Peak work load (watt/kg)	1.0± 0.4	1.1± 0.3	1.4± 0.5	0.1± 0.3	0.26± 0.34	0.36± 0.4	0.910± 0.3	1.089± 0.3	1.206± 0.34	0.19± 0.2	0.069± 0.17	0.25± 0.18	< 0.01	0.316	0.570
PeakVO_2 (_ml/kg/min)	12.8± 5.6	13.3± 3.5	15,8± 4.7	1.06± 5.0	3.48± 3.6	4.5± 5.5	11.6± 2.9	13.3± 3.5	13.8± 4.7	0.08± 3.6	1.67± 2.7	1.76± 3.7	< 0.01	0.818	0.289
1RM for leg extension (kg)	46.6± 17.5	59.5± 21.5	60.1± 22.2	12.84± 13.1	0.643± 19.9	13.5± 21.4	50.5± 20.9	53.5± 21.1	54.5± 22.2	9.36± 10.0	5.36± 18.7	4± 17.2	0.037	0.321	0.699
Frailty level	Intervention group *n* = 41					Control group *n* = 35					*p*-value				
Frailty level according SPPB score	T0 n, (%)	T1 n, (%)		T2 n, (%)		T0 n, (%)	T1 n, (%)		T2 n, (%)		Time			Group	
Frail	11 (36.7%)	8 (26.7%)		2 (6.6%)		4 (25%)	5 (31.3%)		2 (12.5%)		0.015			0.009	
Pre-frail	6 (20%)	1 (3.3%)		6 (20%)		5 (31.3%)	4 (25%)		8 (50%)						
Robust	13 (43.3%)	21 (70%)		22 (73.3%)		7 (43.8%)	7 (43.8%)		6 (37.5%)						
	Intervention group n = 41					control group n = 35					*p* value				
Frailty level according 5MWT	T0 n, (%)	T1 n, (%)		T2 n, (%)		T0 n, (%)	T1 n, (%)		T2 n, (%)		Time			Group	
Frail	10 (33.3%)	4 (12.12%)		1 (3.3%)		7 (43.8%)	5 (31.3%)		2 (12.5%)		0.006			0.22	
Pre-frail	10 (33.3%)	12 (40%)		12 (40%)		7 (43.8%)	6 (37.5%)		4 (25%)						
Robust	10 (33.3%)	14 (47.9%)		17 (56.7%)		2 (12.5%)	5 (31.3%)		10 (62.5%)						

Abbrevations: *T0* assessment at admittance to CR, *T1* assessment at CR-completion, *T2* assessment at three months after CR-completion *n* number, *M* mean, *SD* standard deviation, *6MWD* six-minute walking distance, *m* meters, *SPPB* short physical performance battery test, *5MWT* five-meter walking test, *m/s* meters per second, *kg* kilograms, *ml* mililiters, *min* minutes, *1RM* one repetition maximu

A sample of 46 (60.5%) patients who had physical frailty assessments in T0, T1 and T2 were included in the analysis. SPPB score and 5MWT indicated a comparable number of patients as being frail in the T2 evaluation performed (SPPB 6.5% vs. 5MWT 8.6%). There were more pre-frail patients according to 5MWT (41.3% vs 29.5%) and also fewer (19.6% vs. 33.7%) compared to SPPB score. Improvement towards better health was seen only in SPPB results; on the other hand, there were still more frail patients in CG according to 5MWT (12.5% vs. 3.3%, respectively). A significant difference between groups was detected at all assessment times, for SPPB and at T0 for 5MWT (Table 3).

## Discussion

The aim of our study was to evaluate the short- and mid-term effect of a specially tailored resistance/balance training in older patients after valve surgery/intervention, specifically the impact on functional capacity, physical performance (primary outcome) exercise capacity, muscular strength and prevalence and/or symptoms of physical frailty. The intervention was provided in addition to the usual care CR exercise program (aerobic endurance, respiratory training) during 20 days of in-patient CR.

Our results demonstrate that the additional resistance and balance training provided in the study was accepted and tolerated in the target group of patients. As a result of the CR participation, functional capacity (6MWT), physical performance (SPPB and 5MWT), exercise capacity (peak work load, peak VO_2_) and muscular strength (1RM) were significantly improved in both groups. An additional benefit from the specially tailored resistance and balance training could not be confirmed. On the other hand, the patients that participated in the IG during CR presented themselves with significantly lower physical frailty levels at mid-term follow up visit. However, given the high number of missing data at this visit, the evidence of these results is questionable.

Currently, there are no generally accepted standards on content, volume and intensity of exercise-based CR after valve surgery/intervention. Aerobic endurance training with low to moderate intensity is recommended as a basic therapy [1, 2] and low to moderate intensity resistance exercise is considered a valuable additional exercise mode [1, 2]. In a randomized pilot study (*n* = 27 after TAVI), combined strength and endurance training over 8 weeks led to a significant increase in the peakVO_2_ (3.7 ml/min/kg) and muscle strength compared to the control group [22]. What is more, studies that compared patients after open-heart surgery and TAVI showed that exercise-based CR leads to comparable effects in both groups on 6MWT [7, 23–25] and peak VO_2_ [23]. Our study demonstrates immediate and significant rehabilitation effect on exercise capacity (Wmax) and 1RM as well as 6MWT, SPPB score and 5MWT. These improvements are sustainable and can still be demonstrated three months after completing the CR. Furthermore, the results revealed significant improvements in VO_2_peak, three months after CR completion. Our results are comparable to those of other phase-II CR studies reporting improvements in 6MWD [12, 24–30] after 3–4 week CR. Other studies show that improvements in VO_2_peak are to be expected in outpatient programs with longer duration [29, 31–34]. The improvements in physical performance did not reveal significant differences between groups.

To our knowledge, no other studies evaluated short or medium-term CR impact on SPPB score or 5MWT exclusively in patients after valve surgery/intervention. However, in frail older adults patients (SPPB score ≤ 8) who participated in exercise-based CR (involving resistance, flexibility, balance exercise) after surgery, Molino-Lova et al. [14] demonstrated significant improvements in SPPB score compared to CG one year after discharge from CR. Furthermore, results from Rengo et al. [15] show patients who were admitted to CR with SPPB score ≤ 8 as experiencing significantly more pronounced improvement in gait speed and leg strength (chair-stand) compared to the overall CR population as a result of participation in combined aerobic/resistance training. Moreover, high-speed resistance exercise participation training for older adults living in a community demonstrably improves their SPPB score [35].

The increasing number of older patients admitted to CR has taught us that the target CR goals and the outcome measures have to be adapted. CR goals for these patients should focus on maintaining mobility, as well as avoiding or reducing frailty. This makes the use of instruments to diagnose frailty, mobility and functional capacity necessary, not only for outcome evaluation, but also and especially to be able to tailor the exercise-based CR program to the patient’s special needs [9–11]. Until now there has been no consensus regarding how, and in which patients, frailty should be measured in CR. There are recommendations to use Edmonton or Clinical frailty scales [9, 36], while other authors criticize these questionnaires because of their lack of sensitivity to detect serial changes [10]. Physical performance tests such as the SPPB and 5MWT are more objective and quantitative, and thus more useful to evaluate short-, mid- and long-term effects of CR on physical frailty [10, 19]. In older patients after TAVI, mobility (TUG) has been demonstrated to be a significant predictor (OR 5.12; 95% CI 1.64–16.01; *p* = 0.005) for all-cause mortality in the first year after TAVI [37].

Until now, only a few studies have evaluated the impact of CR on frailty in patients after valve surgery or intervention**.** We used the SPPB score and 5MWT test to evaluate physical frailty level [10, 19, 20]. Both tests indicated a comparable number of patients to be frail in the three evaluations performed; on the other hand, a comparatively higher number of patients were categorized as pre-frail using the 5MWT and fewer as robust compared to SPPB score. This trend was observed at all three time points in our study. This could be explained by the concerns regarding SPPB score and its ceiling effect for individuals with higher fitness levels [10, 15, 38]. In our study, the physical frailty level improved in both groups during the observation period. Significant intervention effects were only seen in the visit three months after CR completion while using SPPB test for physical frailty evaluation. This may be the result of higher levels of physical activity i.e. performing learned exercises at home and emphasizes the importance of patient education, sustainability and empowerment to increase physical activity [10, 39, 40].

### Study limitations

The trial has several limitations: [1] This was a single-center trial and the results may not be generally applicable to all patients after heart valve surgery or intervention [2]; Patient population was heterogenic as the study included patients after open-heart surgery and TAVI [3]; The results may be affected by the imperfect adherence to the intervention and fewer training sessions fulfilled than originally planned - 6.1 ± 1.2 on average (out of 9, 4) A significant number of drop-outs and missing data cases complicated the statistical analysis and limit study results [5]; Study intervention was not only additional exercises, but also telephone calls. These could have affected physical activity in the IG group and influenced the 3-month study results [6]; The trial was unblinded for patients and staff, although during evaluation of physical tests, all researchers were blinded to the allocation group [7]; Inter-rater and intra-rater reliability testing for CPET was not evaluated.

## Conclusions

Exercise-based inpatient CR improves functional capacity, physical performance, exercise capacity and muscular strength in patients after valve surgery or intervention in the short and medium terms. The additional specially tailored resistance/balance training was accepted and tolerated in the patient cohort. SPPB score and gait speed test were demonstrated to be useful for assessing of frailty screening and outcomes evaluation in a setting of CR. Reduced frailty levels were seen in the intervention group of patients three months after CR completion. However, given the high number of missing data at this visit, the evidence of these results is questionable and further studies are needed to learn more about optimal CR content design and outcome assessment in this patient cohort.

## Data Availability

The datasets used and/or analyzed during the current study are available from the corresponding author on reasonable request.

## Appendix 1

**Table 4:** Exercise training program for both usual care (Control group) and Intervention group

Aerobic endurance training 6 days /week (40 min)	
• Supervised individually tailored group training (6–7 patients) • From low to moderate continuous endurance training on cycle ergometer	• Warm up (< 50% target intensity 2 min, gradually increasing load 1–10 W/min up to target intensity within 5–10 min); • exercise phase (100% of the target intensity (30–50% watt_max_ or 60–70% maximal heart rate (HR_max_)), starting with > 5 min and gradually lengthening up to 30 min); • cool down with gradual reduction of the load within 3 min
Aerobic dynamic gymnastics 5 days/week (30 min)	
• Supervised individually tailored group training (6–7 patients) • From low to moderate intensity exercises*,* BORG ≤13 • 1 min of exercises, 45 s rest • From easy/known exercise - to harder/unknown • Equipment: small gymnastic ball, gymnastic stick, chair	• Stretching and breathing exercises (5 min) for warm up and cool down • Seated march: Raising one knee after another, returning to starting position • Straightening bent elbow while standing in front and bending back • Waist bend to both sides while standing • Knee bends in combination with swinging arms • Side/back steps in combination with arm lift • Marching while standing: from heel lift to gentle march involving arm movement • Heel kicks – toe tap behind opposite heel/heel kick behind
Respiratory muscle training 7 days/week, (15 min)	
• Unsupervised exercises after two individually supervised trainings • Equipment: lung exerciser (Respiprogram, Germany). • 10 repetitions, rest between sessions, repeat few times per day.	• Patient was told to slowly and deeply inhale to raise yellow ball. Than hold breath for at least 5 s and exhale until the ball falls.

## Appendix 2

**Table 5:** Additional resistance and balance training sessions 3 times/week for Intervention group. Supervised individually tailored small group training (3 patients)

First week, intensity BORG 11 and < 30% RM	
Balance training (15 min), • 2 min of exercise • 1 min rest /stretching • From easy/known exercise - to harder/unknown • Closing eyes to increase difficulty	• Sitting with a feet on uneven surface • Standing on uneven surface with one foot, changing feet • Standing with one foot in front of other • Putting the weight from heel to toes and back • walking along a line forward/backward, putting one foot in front of other
Strength training (20–25 min) • 3 min rest after each exercise • 3 sets with 5–10 repetitions, with 30 s rest between sets	• Knee extension with resistance band while sitting, changing legs (90° flexion in knee) • Legs abduction with a resistance band while sitting (90° flexion in knee) • Lifting heels to tiptoe with small weights on ankles while standing • Lifting legs and putting them down while sitting with small weight on each ankle (90° flexion in knee)
Second week, intensity BORG 12–13 and 30–50% RM	
Balance training (15 min), • 2 min of exercise • 1 min rest /stretching • From easy/known exercise - to harder/unknown • Closing eyes to increase difficulty	• Standing with one foot in front of other on uneven surface • Switching feet position on uneven surface • Standing on one foot with/without hand on a back of a chair • walking along a line forward/backward, putting one foot in front of the other
Strength training (20–25 min) • 3 min rest after each exercise • 3 sets with 15 repetitions, with 60 s rest between sets	• Legpress • Abduction of the thigh with weight on the ankle while standing • Lifting heels to tiptoe with small weights on ankles while standing • Stepping up and down a stair with weights on ankles
Third week, intensity BORG ≤15 and 50–60% RM	
Balance training (15 min), • 2 min of exercise • 1 min rest /stretching • From easy/known exercise - to harder/unknown • Closing eyes to increase difficulty	• Standing with one foot in front of other on uneven surface • Switching feet position on uneven surface • Catching a ball while standing on uneven surface • Touching the ground with tiptoes to the different sides
Strength training (20–25 min) • 3 min rest after each exercise • 3 sets with 15 repetitions, with 60 s rest between sets	• Legpress • Abduction of the thigh with weight on the ankle while standing • Adduction of the thigh with weight on the ankle while standing • Squats with sitting on a chair with/without hand on a back of a chair

## Abbreviations

CI: Confidence interval
CG: Control group
CPET: Cardiopulmonary exercise testing
CR: Cardiac rehabilitation
HR: Heart rate
IG: Intervention group
NYHA: New York Heart Association
OR: Odds ratio
RPE: Rate of perceived exertion
SPPB: Short physical performance battery test
TAVI: Transcatheter aortic valve implantation
TUG: Timed up and go test
T0: Assessment before randomization at admittance to CR
T1: Assessment after CR
T2: Assessment 3 months after CR completion
W: Watt
1RM: One repetition maximum
5MWT: 5-m walk test
6MWT: Six-minute walk test
